# Enhancing Efficacy and Quality of Life in Patients with Herpes Zoster Infection in Hairy Cell Leukemia

**DOI:** 10.1155/2024/1575161

**Published:** 2024-02-26

**Authors:** Xiaowei Feng, Yuchen Tao, Qi Hu, Yuanxia Liu, Jizhang Bao, Wenwen Jiang

**Affiliations:** ^1^Department of Hematology, Shanghai Municipal Hospital of Traditional Chinese Medicine, Shanghai University of Traditional Chinese Medicine, Shanghai, China; ^2^Department of Pathology, Shanghai Municipal Hospital of Traditional Chinese Medicine, Shanghai University of Traditional Chinese Medicine, Shanghai, China

## Abstract

Hairy cell leukemia (HCL) is an infrequent and persistent B-cell inert lymphoid leukemia. In this study, we present the case of a 71-year-old female patient with a previous diagnosis of variant HCL who experienced a severe herpes zoster infection leading to an extensive skin eruption. The patient's initial diagnosis of HCL occurred 7 years ago, and she underwent treatment with cladribine, interferon, COP (cyclophosphamide, vincristine, and prednisone), benztropine tablets + clarithromycin dispersible, and ibrutinib. Immune disorders resulting from repeated prior chemotherapy and targeted therapy may potentially precipitate herpes zoster infection. Despite an initial two-week period of unresponsiveness to antivirals and nerve nutrition treatments, the introduction of topical Coptis liquid to the treatment regimen yielded significant efficacy. This case report underscores the potential of Chinese medicine as an adjunct to conventional antiviral therapy in the management of herpes zoster infection in immunocompromised patients. This treatment protocol has the potential to enhance efficacy, enhance quality of life, and serve as a more robust foundation for clinical diagnosis and improved treatments.

## 1. Introduction

Hairy cell leukemia (HCL) is an infrequent, persistent malignancy affecting fully developed B lymphocytes within the postgerminal centers, impacting the bone marrow, peripheral blood, and spleen. HCL constitutes a mere 2-3% of all leukemia cases [1, 2]. Infection is widely recognized as the primary contributor to mortality [1]. The patient under consideration in this report exhibited a medical background of variant HCL and was subjected to prolonged administration of targeted oral medications. Consequently, this led to a compromised immune system, heightening vulnerability to infections. The occurrence of herpes zoster can be attributed not only to the immunodeficiency resulting from bacterial and viral infections due to treatment but also to the underlying primary immune disorder. Hence, the exclusive reliance on antiviral and neurotrophic treatment proves insufficient, necessitating the incorporation of traditional Chinese medicine as a crucial component in the management and prevention of bacterial infectious diseases in contemporary times. In this case, we added Chinese medicine into treating a patient with herpes zoster infection and extensive skin breakdown resulting from HCL. This report aims to elucidate the treatment attributes specific to immunodeficient HCL patients, with the ultimate goal of enhancing the rate of recovery.

## 2. Case Presentation

**Figure 1:**
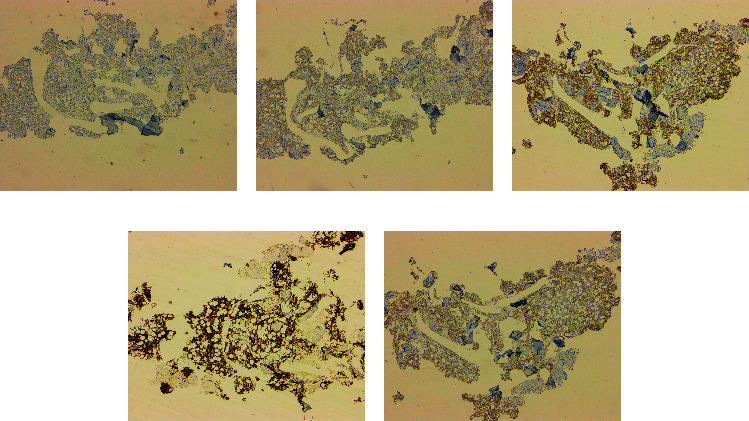
Bone marrow biopsy. (a) CD25 IHC, 4x. (b) CD103 IHC, 4x. (c) CD11c IHC, 4x. (d) CD22 IHC, 4x. (e) CD20 IHC, 4x.

**Figure 2:**
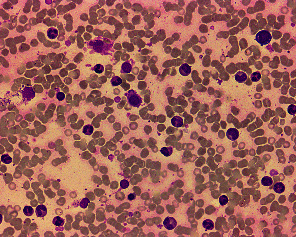
HCL cells in bone marrow smear (wright stain, 40x). HCL cytological features: fried egg-like changes; brush-like protrusions at the edge of the hairy cell envelope.

**Figure 3:**
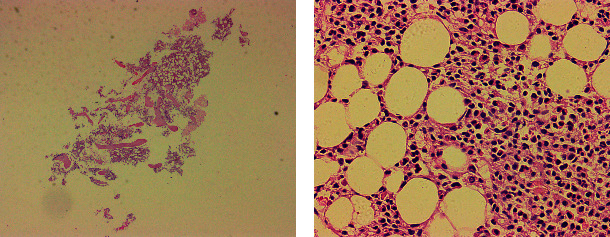
Bone marrow biopsy. (a) H&E, 2x. (b) H&E, 40x.

**Figure 4:**
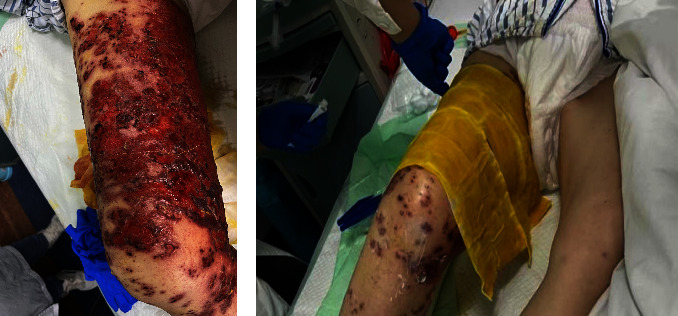
(a) The patient with a large skin ulcer on the right lower extremity with obvious yellowish exudate, high skin temperature, and red skin color with pain. (b) Patients are given Coptis fluid to apply wet on the top of the wound for 30 minutes.

**Figure 5:**
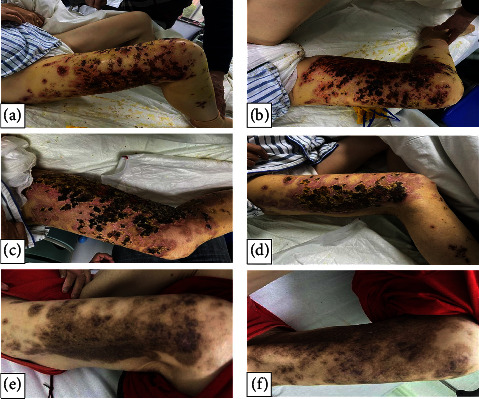
(a–f): Pictures of the patient after treatment: (a, b) 8 days of treatment; (c, d) 13 days of treatment; (e, f) 3 months follow-up.

In 2016, a 71-year-old female patient sought medical attention at the hospital due to the onset of severe pain and distention in the left upper abdomen. Concurrently, the patient experienced pronounced malaise and a weight loss of 7.5 kg over a six-month period. A computed tomography scan confirmed the presence of significant splenomegaly, while blood tests indicated a lymphocyte count of 5.2 × 10^9^/L and a lymphocyte ratio of 57%. The laboratory tests indicated a higher level of bone marrow proliferation, with hair cells comprising 20.5% and lymphocytes accounting for 16.5%. The gross cell immunophenotypes were identified as CD25+, CD103+, CD11c+, CD20+, CD19+, and CD22+ (Figure 1). The bone marrow smear and the peripheral blood smear exhibited the presence of burr-like protuberances. In addition, the bone marrow biopsy revealed evidence of active hyperplasia, with certain tumor cells displaying a distinct “fried-egg” morphology characterized by well-defined cell membranes and copious clear cytoplasm (Figure 2). The bone marrow trephine biopsy additionally substantiated the extensive infiltration of the marrow (Figure 3), concomitant with the presence of the BRAFV600E gene mutation. Consequently, the patient was diagnosed with variant hairy cell leukemia. Over the course of two years, the patient underwent multiple cycles of initial treatment with cladribine, resulting in partial remission. However, relapse occurred after one year, characterized by splenomegaly and a decline in the blood cell count. Subsequent retreatment with cladribine proved to be ineffective. The patient was initiated on a new treatment regimen consisting of interferon, COP (cyclophosphamide, vincristine, and prednisone) administered three times, and benztropine tablet + clarithromycin dispersible tablet regimen also administered three times. Despite this treatment, the patient's spleen continued to enlarge progressively, indicating disease progression. However, in March 2019, after switching to oral ibrutinib, there was a significant reduction in spleen size. Flow cytometry analysis revealed minimal residual disease (MRD), characterized by a significant decrease in CD5^−^CD10^−^ monoclonal B cells, suggesting partial remission of the disease. Therefore, it can be concluded that the leukemia was partially resolved. On November 14, 2022, the patient exhibited the emergence of a substantial herpes zoster rash accompanied by notable skin ulceration on the right lower extremity. The patient received treatment at a different medical facility, which involved the administration of adenosine cobalamin for nerve nutrition and valaciclovir as an antiviral agent; however, the patient's response to this treatment was unsatisfactory. On November 28, 2022, the patient sought medical attention at our hospital, presenting with a sizable skin ulcer on the right lower extremity that displayed evident yellowish exudate, elevated skin temperature, red coloration, and pain (Figure 4(a)). The patient was administered oral mecobalamin for nerve nutrition, oral valaciclovir, and cefepime + linezolid for the treatment of infection, as indicated by wound swab testing which suggested the presence of Staphylococcus aureus infection. In addition, topical treatment was provided by applying Coptis liquid through a wet compress on the wound for a duration of 30 minutes (Figure 4(b)). Subsequently, the wound was left exposed and devoid of moisture. This procedure was performed three times daily, with each instance involving the cleansing of the wound using sterile saline and subsequent disinfection with iodophor prior to Coptis treatment. Following 8 days of this therapeutic regimen, the patient persisted in experiencing discomfort, accompanied by a slightly elevated skin temperature. However, there was a reduction in exudate production, and the affected skin exhibited a crust formation that was more limited in extent compared to previous observations (Figures 5(a) and 5(b)). Based on the aforementioned treatment plan, the crusted area was subjected to LongZhuRuangao application to alleviate symptoms such as swelling, pain, decay, and muscle impairment. Following a 13-day treatment period (Figures 5(c) and 5(d)), the patient's initial extensive ulcerated skin surface crust and exudates had substantially diminished, accompanied by a reduction in skin temperature. After a subsequent 3-month follow-up, the skin crusts had completely resolved (Figures 5(e) and 5(f)).

## 3. Discussion

The age of onset for HCL typically falls within the range of 42 to 62 years, with a prevalence that is 4-5 times higher in males than in females [3, 4]. The initial course of treatment typically encompasses cladribine, pentostatin, and interferon, all of which demonstrate notable efficacy. Nevertheless, it is important to note that approximately 50% of patients remain susceptible to relapse [5]. Hence, alongside the aforementioned medications, patients experiencing relapsed and refractory hairy cell leukemia (HCL) are administered drugs that specifically target the pathogenic pathways of HCL, including BRAF inhibitors (vemurafenib and dabrafenib), Bruton tyrosine kinase (BTK) inhibitors (ibrutinib), and anti-CD22 immunotoxins (moxetumomab pasudotox) [6]. Repeated administration of targeted drugs for the purpose of managing a high rate of disease recurrence can result in the manifestation of severe adverse effects, notably prolonged immunosuppression, thereby rendering individuals more susceptible to infections.

Infection is the predominant etiology of morbidity and mortality among individuals diagnosed with HCL. Research has demonstrated that the incidence of infection as a comorbidity in HCL patients is approximately 30%, with a subset of 10–20% manifesting cutaneous infections [7]. Bacterial and viral infections, specifically herpes zoster, are prevalent among individuals. Varicella-zoster virus (VZV), a distinctive member of the herpesvirus family, solely resides in humans, and consequently, the disease exclusively affects humans. The primary infection presents as chickenpox, followed by viral latency within the ganglia. In instances where the individual's immune system is compromised, the dormant virus within the ganglia reactivates, resulting in the development of herpes zoster or shingles. The manifestation of the herpes rash typically occurs in the dermatomes corresponding to the innervation of the nerves, primarily in the thoracic region, subsequently extending to the lumbar, cervical, cranial, and sacral regions. Noteworthy complications associated with this infection encompass postherpetic neuralgia, motor paralysis, and ocular herpes zoster [8–10]. The infection typically presents with localized cutaneous symptoms; nevertheless, immunocompromised patients may exhibit involvement of internal organs and other secondary infections [11–13]. The research findings indicate a correlation between the severity of VZV infection and the nature of the primary disease, whether it is benign or malignant, with notable distinctions. Individuals with malignant primary diseases, such as hematological cancers, may face an elevated susceptibility to VZV infection, potentially attributable to the necessity of undergoing multiple chemotherapy treatments that result in pronounced immunodeficiency. In individuals afflicted with disseminated herpes zoster, malignant tumors, or severe immunocompromisation, the viral dissemination occurs via the bloodstream, leading to extensive cutaneous eruptions resembling varicella, excluding the affected area. Concurrently, systemic toxic manifestations, including elevated body temperature, frequently manifest alongside the infection.

In a study comprising 113 patients diagnosed with hairy cell leukemia (HCL) and presenting with cutaneous infections, the prevailing occurrence was observed to be herpetic lesions, encompassing herpes simplex and herpes zoster widespread varicella. Conversely, the incidence of pemphigus, dermatophytes, Candida infections, and sepsis (including abscesses, cellulitis, folliculitis, and pyoderma) was comparatively less frequent [14]. Therefore, the administration of various chemotherapeutic agents and the compromised immune response observed in individuals with a primary malignant neoplasm can augment the probability of herpes zoster infections. Furthermore, a comprehensive analysis elucidating the dermatological alterations in HCL revealed that the utilization of antileukemic medications in HCL patients renders them more vulnerable to opportunistic cutaneous infections caused by viral, fungal, and bacterial pathogens [15]. VZV infections are commonly observed following stem cell transplantation, and research has demonstrated that in the absence of prophylactic administration of antiviral medications, a substantial proportion of patients (ranging from 20% to 53%) experience VZV infection within the first year posttransplantation [16–18]. Hence, it is strongly advised to administer preventive antiviral therapies, such as acyclovir, within the first year following allogeneic hematopoietic stem cell transplantation, as this measure has been shown to effectively diminish the occurrence of herpes zoster [19]. In this particular instance, the patient diagnosed with primary hematologic malignancy encountered relapse despite undergoing multiple rounds of chemotherapy and targeted therapy, leading to an immunodeficient condition comparable to severe immunodeficiency observed in transplant recipients. Consequently, the administration of prophylactic antiviral medication subsequent to repeated chemotherapy can effectively mitigate the likelihood of herpes zoster occurrence.

Currently, a plethora of treatment alternatives for herpes zoster exists both domestically and globally. The current medicine predominantly encompasses antiviral medications, anti-inflammatory agents, and nerve-nourishing therapies [20]. The patient described in this report received appropriate treatment for her primary disease at an early stage but unfortunately experienced a poor outcome. Upon presentation to our hospital, the herpes breakout was still in the generalized stage. Research has demonstrated that herpes zoster can lead to systemic disseminated infection, manifesting as blisters, erythematous papules, vesicles, or pustules, and involvement of organs through blood-borne transmission, such as encephalitis, pneumonia, and hepatitis. The mortality rate associated with this condition has surpassed 50%, significantly impacting the patient's quality of life [21–23].

The patient under study utilized a liquid, which is formulated with the Chinese herb Rhizoma Coptidis. Coptidis, being a traditional Chinese herbal medicine, has been documented to possess antibacterial, antioxidant, antihyperglycemic, and anti-inflammatory properties [24, 25]. Berberine, a prominent compound found in the Chinese herb Rhizoma Coptidis, was the focus of this study. In current research, the researchers successfully isolated six new alkaloids (coptisine A–F, 1–6) and 26 previously identified ones (7–32) from the rhizomes of *C. chinensis*. In addition, we conducted an evaluation of the inhibitory properties of these compounds against acetyl cholinesterase (AChE). Contemporary pharmacological studies have revealed that Coptis demonstrates a wide range of antibacterial effects against strains such as *S. aureus* and *Streptococcus hemolyticus* [26]. It is revealed that berberine found in Coptis exhibits protective properties against cytomegalovirus-induced apoptosis in spiral ganglion cells. In vitro experiments demonstrated that Coptis aqueous decoction displayed inhibitory effects on *S. aureus* in antibacterial assays. Alkaloids were identified as the primary contributors to the antibacterial and antiviral effects, while the remaining components exhibited limited efficacy. The patient in our study employed the direct application of Coptis liquid onto the skin affected by extensive blisters and exudates. Specifically, the Coptis liquid was administered onto gauze, which was subsequently placed upon the wound for a duration of 30 minutes, without any covering. This approach successfully facilitated the absorption of exudate and safeguarded the nociceptive receptors within the wound, thereby resulting in alleviation of pain. The application of Coptis liquid resulted in the formation of a protective layer on the wound, effectively shielding it from external factors and impeding the proliferation and infiltration of bacteria. In addition, this intervention expedited the process of liquefaction and elimination of infected tissues through reparative mechanisms, enhanced microcirculation, and alleviated tissue hypoxia, ultimately expediting the healing process of the ulcerated surface.

## 4. Conclusions

This report presents a case study of a female patient with HCL who experienced severe disseminated herpes zoster infection in the context of recurrent primary disease, immunodeficiency resulting from long-term chemotherapy, and targeted drug therapy. Despite two weeks of ineffective antiviral and nerve-nourishing treatment, a significant improvement was observed when internal and external Chinese medicines were combined. Hence, in instances of disseminated herpes zoster among immunocompromised individuals, the utilization of topical and external treatments alongside oral antiviral medications can effectively mitigate exudation and facilitate cutaneous regeneration. The management of immune deficiency may involve the implementation of vaxilovir-based antivirals as a prophylactic measure.

## Data Availability

The original contributions presented in the study are included in the article, and further inquiries can be directed to the corresponding author.
